# The effect of acetaminophen on ubiquitin homeostasis in *Saccharomyces cerevisiae*

**DOI:** 10.1371/journal.pone.0173573

**Published:** 2017-03-14

**Authors:** Angelina Huseinovic, Jolanda S. van Leeuwen, Tibor van Welsem, Iris Stulemeijer, Fred van Leeuwen, Nico P. E. Vermeulen, Jan M. Kooter, J. Chris Vos

**Affiliations:** 1 AIMMS-Division of Molecular Toxicology, Department of Chemistry and Pharmaceutical Sciences, VU University Amsterdam, Amsterdam, The Netherlands; 2 Donnelly Centre for Cellular and Biomolecular Research, University of Toronto, Toronto, Ontario, Canada; 3 Division of Gene Regulation, Netherlands Cancer Institute, Amsterdam, The Netherlands; 4 AIMMS-Department of Molecular Cell Biology, Section Genetics, VU University Amsterdam, Amsterdam, The Netherlands; Louisiana State University Health Sciences Center, UNITED STATES

## Abstract

Acetaminophen (APAP), although considered a safe drug, is one of the major causes of acute liver failure by overdose, and therapeutic chronic use can cause serious health problems. Although the reactive APAP metabolite N-acetyl-p-benzoquinoneimine (NAPQI) is clearly linked to liver toxicity, toxicity of APAP is also found without drug metabolism of APAP to NAPQI. To get more insight into mechanisms of APAP toxicity, a genome-wide screen in *Saccharomyces cerevisiae* for APAP-resistant deletion strains was performed. In this screen we identified genes related to the DNA damage response. Next, we investigated the link between genotype and APAP-induced toxicity or resistance by performing a more detailed screen with a library containing mutants of 1522 genes related to nuclear processes, like DNA repair and chromatin remodelling. We identified 233 strains that had an altered growth rate relative to wild type, of which 107 showed increased resistance to APAP and 126 showed increased sensitivity. Gene Ontology analysis identified ubiquitin homeostasis, regulation of transcription of RNA polymerase II genes, and the mitochondria-to-nucleus signalling pathway to be associated with APAP resistance, while histone exchange and modification, and vesicular transport were connected to APAP sensitivity. Indeed, we observed a link between ubiquitin levels and APAP resistance, whereby ubiquitin deficiency conferred resistance to APAP toxicity while ubiquitin overexpression resulted in sensitivity. The toxicity profile of various chemicals, APAP, and its positional isomer AMAP on a series of deletion strains with ubiquitin deficiency showed a unique resistance pattern for APAP. Furthermore, exposure to APAP increased the level of free ubiquitin and influenced the ubiquitination of proteins. Together, these results uncover a role for ubiquitin homeostasis in APAP-induced toxicity.

## Introduction

Acetaminophen (N-acetyl-p-aminophenol, paracetamol, APAP) is a widely used over-the-counter drug and is considered a safe analgesic and antipyretic at therapeutic doses. An overdose, however, induces hepatotoxicity and is one of the major causes of acute liver failure in the USA and Western Europe [1]. Liver toxicity is in part due to the chemically reactive APAP metabolite N-acetyl p-benzoquinoneimine (NAPQI), which is generated by cytochrome P450 [2,3]. Susceptibility to hepatotoxicity by APAP may increase by concurrent medications, poor nutritional status, chronic alcohol abuse, obesity and non-alcoholic fatty liver disease [4,5].

APAP can already cause toxicity at therapeutic doses. For example, it was identified as a risk factor for acute renal injury [6] and it can cause onset of Stevens-Johnsons syndrome and toxic epidermal necrolysis [7,8]. Epidemiological studies revealed a link between the use of APAP during pregnancy and the incidence of attention deficit hyperactivity disorder (ADHD) and hyperkinetic disorder in children [9]. Another study showed a link between APAP and asthma [10] and long term-use was associated with increased incidence of cancer [11,12].

Although NAPQI formation by cytochrome P450 is considered the major cause of liver and renal toxicity, toxicity can occur in its absence. For example, Miyakawa et al. (2015) showed cytochrome P450 independent toxicity in mouse hepatocytes, especially at higher APAP concentrations [13], while Jensen et al. (1996) reported toxicity with and without expression of cytochrome P450, which in both cases caused induction of spindle disturbances in Chinese hamster V79 cells [14]. Srikanth et al. (2005) observed APAP toxicity in yeast in the absence of NAPQI-derived metabolites, while overexpression of ABC-transporters Snq2 and Flr1 resulted in increased drug resistance [15]. Furthermore, para-aminophenol, another APAP metabolite, produced by an N-acetyltransferase, can also result in toxicity [16]. It is therefore possible that other reactive metabolites are formed or yet unknown mechanisms of APAP-induced toxicity exist [17].

The budding yeast *Saccharomyces cerevisiae* is frequently used to study drug-induced toxicity [18,19]. Although it is a simple eukaryotic cell with approximately 6000 genes, many essential pathways are highly conserved between human and yeast. Furthermore, the availability of a complete non-essential gene deletion library allows for a relatively straightforward screening of genes and pathways that are involved in drug-induced toxicity [18]. Chemical-genomic screens in yeast have revealed a wealth of information about the various mechanisms of drug action and have identified novel functions for genes [20], some of which are relevant to human biology [21]. Successful examples for the identification of the mode of action of drugs derived from screening the library of yeast deletion strains are arsenic [22], cisplatin [23], and quinine [24].

The genes that contribute to APAP sensitivity or resistance are largely unknown. Therefore, we decided to perform a genomic screen in yeast to study APAP-induced toxicity. By varying the concentration of APAP and growth temperature, we identified genes relevant for APAP sensitivity in diverse cellular processes, such as RNA polymerase II transcription, chromatin remodeling, and protein and vesicle-mediated transport. The most striking observation, however, is the crucial role of ubiquitin-mediated processes, whereby ubiquitin deficiency results in APAP resistance.

## Materials and methods

### Chemicals and stock solutions

All chemicals were purchased from Sigma (St. Louis) at high purity except for benomyl (Santa Cruz Biotechnology). Yeast extract and peptone were obtained from Melford, and yeast nitrogen base, glucose and amino acids were obtained from Sigma.

### Strains and media

Haploid deletion strains of *Saccharomyces cerevisiae* in the BY4741 background (*MATa; ura3Δ0; leu2Δ0; his3Δ1; met15Δ0; geneΔ*::*kanMX4*) were obtained from EUROSCARF (Frankfurt, Germany). A specialized, selective collection of 1522 strains contained mainly haploid deletion strains as well as 71 DamP strains (decreased abundance by mRNA perturbation) related to nuclear processes, like DNA damage and chromatin remodeling [25]. For the purpose of the toxicity screen and the spot dilution assays the yeast strains were grown in YPD medium (1% yeast extract 2% peptone 2% glucose and 2% agar for plates) or in selective SC-URA medium (0.67% yeast nitrogen base, 2% glucose and essential amino acids) and spotted on YPD medium containing different concentrations of APAP (0, 50, 60, 70, 80, 90 and 100 mM).

### Plasmids and transformation

All genes were cloned by PCR amplification of a chromosome region containing ~900 bp upstream and ~500 bp downstream of the coding region of the corresponding gene using BY4741 DNA as the template. The PCR products of *UBI4* used for the overexpression experiments were cloned into a multi copy vector Yeplac195 and the PCR products used for the complementation assay were cloned into the single copy vector Yeplac33. The clones were identified by restriction digestion and additionally sequenced to verify the correct DNA sequence. The primers used will be made available upon request. The plasmid was transformed into yeast cells by the freeze-thaw method as previously described [26].

### Focused APAP toxicity screen

A focused collection of 1522 yeast mutants involved in nuclear processes [25] was used to screen for genes involved in APAP induced toxicity. The screen was performed by pinning the yeast strains in a 24x36 colony format using a RoToR replicator (Singer Instruments) on YPD plates containing YPD medium with different concentrations of APAP: 0, 50, 60, 70, 80, 90 and 100 mM. The plates were pinned and incubated at 30°C and 37°C and photographed each day with a digital camera (Canon Powershot G5, 5.0 megapixels). Photos were converted to greyscale in Photoshop (Adobe) and saved as a.tiff file. Cell Profiler [27–29] was used to quantify colony size as pixel area. For each colony, the integrated intensity (colony size + intensity) and the area (colony size) were exported to Excel.

Sensitive strains were identified using the 70 mM and 60 mM APAP plates, at 30°C or 37°C, respectively, as at these conditions only sensitive strains exhibited a growth restriction when compared to the wild type cells. For each mutant, the ratio in colony size (expressed as pixel area) between control (no APAP) and 60 mM APAP plates was calculated, to correct for differences in nutrient availability and fitness defects of some mutants at standard conditions. Hits were defined as those strains showing an APAP/control ratio < 0.4 (S2 Table). Strains that had a pixel area below 130 on the control plate were considered slow growers and excluded from the analysis. Strains sensitive at 37°C, but not at 30°C, were identified with the following additional criteria. (1) They exhibited normal growth at 30°C and 37°C on the control plates without APAP (pixel area above 130) and the pixel area ratio between 37°C and 30°C plates without APAP was > 0.85 (in order to avoid temperature sensitive mutants). (2) They were growing normally on the plates containing 70 mM APAP at 30°C (pixel area ratio between 70 mM and 0 mM APAP was > 0.75); they were only sensitive at plates containing 60 mM APAP at 37°C (pixel area ratio between 37°C 60 mM and 30°C 70 mM, and ratio between 37°C 60 mM and 0 mM were < 0.5). The experiments at 37°C were performed in duplicate and only the strains that showed a consistent growth decrease in both duplicate plates were taken into account. The 30°C incubations were performed as single experiments, therefore, only the strains that were sensitive in a dose-dependent manner for the conditions 0, 50, 60, 70 and 80 mM APAP were taken into account. Resistant strains were determined from the plates containing 80 mM APAP that were incubated at 37°C. The strains that showed increased growth, measured as the colony (pixel) area compared to the wild type on both duplicate plates, were taken into account. Subsequently, resistant strains were rescreened by performing a spot dilution assay and strains that exhibited higher resistance, measured as a higher growth rate when compared to the WT, were considered hits.

### Confirmation of APAP resistance by spot dilution assay

The identified APAP resistant mutants were isolated from the control plates of the screen and grown overnight in YPD medium at 30°C. The cultures were subsequently diluted to an OD_600_ = 0.05 and 4 additional 5-fold serial dilutions were made. The cells were spotted on YPD agar plates with or without APAP using a 96-well replica plater (Sigma). Plates were imaged daily for three days. The APAP resistance of each strain was determined using a semi-quantitative analysis by visually comparing the growth rate to the WT cells.

### Gene Ontology (GO) enrichment

Lists of APAP-resistant and -sensitive mutant genes were analyzed further for biological processes enrichment using the online GO enrichment analysis tool provided by *Saccharomyces* Genome Database (SGD). For each biological process, enrichment for genes with altered APAP sensitivity when compared to the complete set of 1522 genes on our array was calculated. Enrichment of sensitive and resistant strains was performed with and without Holm-Bonferroni correction, respectively, and only enrichments with a p-value <0.05 were considered significant.

### Complementation assay

A single copy *URA3* plasmid containing the expression cassette of a gene of interest was transformed into the corresponding deletion strain. An empty plasmid was used as a negative control. The cultures were grown in SC-URA medium overnight at 30°C and a spot dilution assay was subsequently performed as described above.

### Ubiquitin-level analysis by Western blotting

For the determination of ubiquitin levels after APAP exposure, the overnight cultures (16h) were grown at 30°C and subsequently diluted to OD_600_ = 0.5. Prior to APAP exposure, the cultures were pre-incubated at 37°C for 3 hrs. Subsequently, the cells were treated with 0, 25 or 50 mM APAP for 2 h, harvested by centrifugation and frozen at -20°C. Protein extracts were made using a trichloroacetic acid precipitation protocol as described before [30] and the proteins were dissolved in Laemmli buffer without bromophenol blue (50 mM Tris-HCl pH 6.8, 2% SDS, 10% glycerol, 1% β-mercaptoethanol, 12.5 mM EDTA supplemented with 2.5 mM NEM, 1 mM PMSF, 10 mM DTT and protease inhibitor). The samples were diluted 1:40 in water and the amount of total protein was determined by a Protein assay (Bio-Rad). Subsequently, bromophenol blue was added to the samples to a final concentration of 0.02% and a Western blot was performed. Briefly, samples were loaded on 10% bis-Tris gels and the proteins were separated in MES buffer (Life technologies). The proteins were blotted on a 0.2 μm PVDF membrane (Thermo scientific). The membrane was blocked in blocking solution purchased from Li-Cor. Ubiquitin antibody (P4D1, mouse monoclonal) used for detection of total ubiquitin was ordered from Santa Cruz (sc-8017), and anti-actin antibody was ordered from Merck Millipore (MAB1501). The secondary antibodies were IRDye® 680RD Goat anti-Mouse (Li-Cor, 925–68070) and IRDye® 800CW Goat anti-Mouse (Li-Cor, 926–32210). The detection was performed using an Odyssey imager.

## Results

### Identification of deletion mutants resistant to APAP

Initially, a genome-wide screen for resistant non-essential gene deletion mutants was performed to identify genes that are important for APAP toxicity (S1 Text and S1 Table). The screen was designed to identify APAP resistant strains with the aim to isolate a mutant unable to metabolize APAP into para-aminophenol, which is a toxic metabolite of APAP and could be responsible for the observed APAP toxicity. This screen was performed at 100 mM APAP and 37°C because WT yeast cells do not grow under these conditions (WT yeast cells do grow at 100 mM APAP and 30°C). The screen identified several resistant mutants but interestingly, none of them involved a gene encoding a drug-metabolizing enzyme. Instead, in several mutants genes were defective that are involved in the DNA damage response pathway (DDR), including *UBC13* and *RFX1*. Ubc13 forms a heterodimeric complex with Mms2 and acts as an E2 ubiquitin conjugating enzyme. Together with the E3 ubiquitin ligase Rad5, it polyubiquitinates proliferating cell nuclear antigen (PCNA) during post-replication DNA repair [31,32].

**Fig 1:**
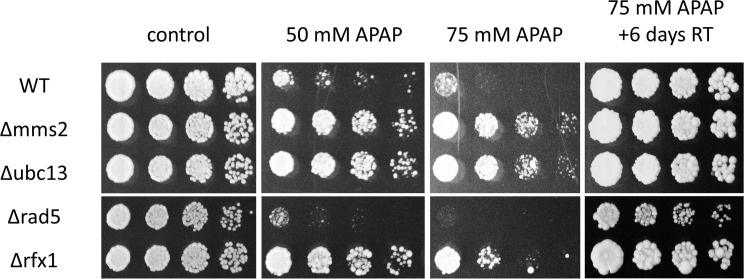
Deletion of DDR genes *MMS2*, *UBC13*, and *RFX1* confers resistance to APAP. Fivefold serial dilutions of a cell suspension of the mutant strains with optical density OD_600_ = 0.05 were plated on YPD plates containing 0, 50 and 75 mM APAP and incubated for one (control) and three days at 37°C. After three days, the 75 mM plate was transferred to room temperature (RT) for 6 days. The strains used were WT (BY4741), *Δmms2*, *Δubc13*, *Δrad5 and* *Δrfx1*.

We performed a spot dilution assay with WT, Δ*mms2*, Δ*ubc13*, and Δ*rfx1* strains, as well as *Δrad5*, in order to confirm resistance and to test toxicity at lower APAP concentrations. Δ*mms2*, Δ*ubc13*, and Δ*rfx1* already have a substantial growth advantage at 50 mM APAP when compared to WT (Fig 1). In contrast, *Δrad5* shows a higher sensitivity towards APAP than WT. PCNA ubiquitination by Mms2/Ubc13 and Rad5 is thus probably unrelated to APAP resistance. When the plates were transferred to room temperature, growth of the WT and Δ*rad5* resumed, indicating a temperature- and APAP-dependent, and reversible growth arrest (Fig 1). A cell-cycle specific growth arrest was not observed (data not shown).

### Detailed screen of 1522 nuclear mutant strains

The unexpected identification of DDR as a potential pathway contributing to APAP toxicity prompted us to concentrate on 1522 genes involved in nuclear processes, instead of APAP metabolism. This more selective screen was based on a study that was focused on DNA repair and chromatin remodeling [25]. The reduced number of strains allowed us to use two different temperatures (30°C and 37°C) and a range of APAP concentrations (50, 60, 70, 80, 90 and 100 mM) to differentiate between more resistant and sensitive growth phenotypes. The selective library of 1522 mutant strains was comprised of 1451 non-essential gene deletion strains and 71 DAmP strains of essential genes (which affect mRNA stability through disruption of the 3’-UTR [33]). Included in this library were *Δmms2* and *Δubc13*, which served as positive controls. The complete list of these strains is presented in S2 Table.

From the comprehensive screening of this collection, 140 mutants were identified showing tolerance to 80 mM APAP at 37°C. By performing spot dilution assays, 107 out of 140 strains were confirmed to be resistant to APAP (S3 Table). The resistant strains included *Δubc13 and Δmms2; Δrfx1* was not present in the library.

Apart from mutants more resistant than WT, also mutants that showed enhanced sensitivity towards APAP were identified. We classified two groups of APAP sensitive strains: 73 very sensitive strains, which were consistently growing more slowly or not growing at all at lower APAP concentrations (50, 60 and 70 mM) and lower temperature (30°C) when compared to WT (S4 Table), and a group of 53 sensitive strains that were only sensitive when incubated at 37°C, 60 mM APAP (S5 Table).

To confirm the involvement of the identified genes in APAP toxicity, complementation assays were performed for a subset of the strains, namely those involved in DNA damage repair and ubiquitin recycling and homeostasis: Δ*mms2*, Δ*ubc13* and Δ*doa1*. For all three APAP resistant strains, expressing the corresponding genes from a single copy plasmid complemented the APAP resistant phenotype, making the level of resistance comparable to the WT. These results confirmed that indeed, the APAP resistance was due to the deletion of any of these genes (S1 Fig).

Furthermore, we selected a set of deletion strains highly sensitive to APAP at 30°C, namely Δ*htz1*, Δ*gcn5*, Δ*swr3* and Δ*vps71* to test for complementation. Expression of these four genes from a single copy plasmid in the corresponding deletion strain restored growth at 70 mM APAP, showing that loss of these genes is indeed responsible for increased APAP sensitivity (S2 Fig).

### GO enrichment analysis of APAP resistant and sensitive strains

GO enrichment analysis was performed on the 107 APAP resistant strains to identify the biological processes involved in the toxicity (Table 1). The GO analysis revealed protein ubiquitination, mitochondria-nucleus signaling, and RNA polymerase II transcription as important processes in APAP toxicity in yeast.

**Table 1:** APAP resistant and sensitive strains and GO enrichment of biological processes, presented in alphabetical order.

**Resistant strains.**		
107 out of 1522	*AAD4*, *AIR2*, *ALP1*, ***APC4***, *ARP1*, *BMH2*, ***CDC23***, ***CDC34***, ***CDC36***, *CLB3*, *CMP2*, *CPA1*, *CUL3*, *DAL81*, *DOA1*, *DOM34*, *EMP70*, ***ESS1***, *FUN30*, *GAC1*, *GFD1*, *GFD2*, *GIS2*, *GNP1*, *GRX4*, *GSH2*, *HAT1*, *HCR1*, *HNT1*, *HRD1*, *HSM3*, *ISY1*, *IXR1*, *JEM1*, *JHD2*, *JNM1*, *KAR5*, *LOS1*, *MBP1*, ***MED6***, *MKS1*, *MLP2*, *MMS2*, ***MPE1***, *MTC7*, *NAM7*, *NHP6A*, *NMD2*, ***NUT2***, *PAC1*, *PET18*, *PSH1*, *PSY4*, *RAD51*, *RAD59*, *RAS2*, RGR1, *RIM20*, ***RPB5***, ***RPB7***, *RPL16A*, *RPL8B*, *RPS29A*, ***RSP5***, *RTG1*, *RTG2*, *RTG3*, *RTT10*, *SAK1*, *SAP185*, *SCJ1*, *SGN1*, *SIR1*, *SIW14*, *SKG1*, *SKN7*, ***SRB4***, *STP4*, ***TAF12***, ***TAF7***, ***TAF8***, *TAN1*, *TCO89*, *TDA3*, ***TFB3***, ***TFB4***, *THR1*, *TOF1*, *TOM7*, *TPM2*, *TSR2*, *TSR3*, *UBC11*, *UBC13*, *UBI4*, *UBP11*, *UCC1*, *UPF3*, *URN1*, *YCK3*, *YER077C*, *YGL081W*, *YNG1*, *YNR065C*, *YPL041C*, *YSY6*	
GO term	Genes	p-value
Protein ubiquitination (18 out of 101)	***APC4***, *BMH2*, ***CDC23***, ***CDC34***, ***CDC36***, *CUL3*, ***ESS1***, *HRD1*, *MMS2*, ***MPE1***, *NAM7*, *PSH1*, ***RSP5***, *UBC11*, *UBC13*, *UBI4*, *UCC1*, *UPF3*	8,09E-05
Transcription initiation from RNA polymerase II promoter (8 out of 33)	***ESS1***, ***MED6***, *NHP6A*, ***RPB7***, ***SRB4***, ***TAF12***, ***TAF7***, ***TAF8***	1,20E-03
Mitochondria-nucleus signaling pathway (4 out of 5)	*MKS1*, *RTG1 RTG2*, *RTG3*	9,98E-05
**Sensitive strains at 30°C**		
72 out of 1522	*ADA3*, ***ARP4***, *ARP6*, *ARV1*, *BEM1*, *BUB1*, *BUB3*, *BUD27*, *CCS1*, *CSF1*, *CTK3*, *DBP7*, *DEG1*, *DST1*, *ERG3*, *ERV14*, ***ESA1***, *FEN1*, *FKS1*, *GAS1*, *GCN5*, *GCR2*, *GET2*, *GRR1*, *GUP1*, *HTZ1*, *IPK1*, *KEX2*, *KRE1*, *LEM3*, *LSM1*, *LSM6*, *MDM34*, ***MED7***, *MNN10*, *MRE11*, ***ORC1***, *PHO80*, *POP2*, *RHO4*, ***RPB3***, *RPB9*, ***RSC3***, *SAC1*, *SAC3*, *SGF73*, *SHE4*, *SMI1*, *SPT3*, *SRB2*, *SSD1*, ***STH1***, *SUR4*, *SWA2*, *SWC3*, *SWC5*, *SWI3*, *SWR1*, *TEF4*, ***TFB1***, ***TFG1***, *TFP1*, *TOP1*, *TPS1*, *TPS2*, *VMA21*, *VPS1*, *VPS51*, *VPS53*, *VPS71*, *VPS72*, *YAF9*	
GO term	Genes	p-value
Protein-DNA complex subunit organization (17 out of 86)	***ARP4***, *ARP6*, *BUB1*, *DST1*, ***ESA1***, ***ORC1***, ***RSC3***, *SGF73*, *SRB2*, ***STH1***, *SWC3*, *SWC5*, *SWR1*, ***TFG1***, *VPS71*, *VPS72*, *YAF9*	9.11E-04*
Chromatin remodeling (12 out of 58)	***ARP4***, *ARP6*, *HTZ1*, ***RSC3***, ***STH1***, *SWC3*, *SWC5*, *SWI3*, *SWR1*, *VPS71*, *VPS72*, *YAF9*	6.60E-03*
**Sensitive strains at 37°C**		
53 out of 1522	*ARL1*, *ARO1*, *ASC1*, ***CDC28***, *CIK1*, *COG5*, *COG6*, *COG7*, *COG8*, *CPR6*, *CWH41*, *ENV11*, *ESC2*, *FPK1*, *GTR1*, *IDH1*, *INP53*, *IWR1*, *LST4*, *MEH1*, *NCL1*, *OPI3*, *PDC1*, *PMP3*, *PMT5*, *RAV2*, *RDI1*, *RIC1*, *ROT2*, *RPL19A*, *RPS11A*, *RPS4A*, *RPS8A*, *RRD1*, *SAT4*, *SEC22*, *SGF73*, ***SMT3***, *SNX4*, ***TAF9***, *TMA19*, *TPS1*, *TPS2*, *TRS85*, *VAM3*, *VAM7*, *VID22*, *VPS17 VPS29*, *VPS38*, *VPS41*, *VPS5*, *YPT6*	
GO term	Genes	p-value
Protein transport and establishment of protein localization (24 out of 205)	*ARL1*, *COG5*, *COG6*, *COG7*, *COG8*, *GTR1*, *INP53*, *IWR1*, *LST4*, *MEH1*, *RAV2*, *RIC1*, *SEC22*, *SNX4*, *TRS85*, *VAM3*, *VAM7 VID22*, *VPS17 VPS29*, *VPS38*, *VPS41*, *VPS5*, *YPT6*	6.52E-05*
Vesicle-mediated transport (17 out of 47)	*ARL1*, *CDC28*, *COG5*, *COG6*, *COG7*, *COG8*, *INP53*, *RIC1*, *SEC22*, *SNX4*, *TRS85*, *VAM3*, *VAM7*, *VPS17*, *VPS29*, *VPS41*, *VPS5*, *YPT6*	6.47E-03*
Cytoplasm-to-vacuole targeting (CVT) pathway (8 out of 18)	*COG5*, *COG6*, *COG7*, *COG8*, *SNX4*, *TRS85*, *VAM7*, *VSP41*	2.08E-04*

Note: p values marked with * are calculated with Holm-Bonferroni correction. Only the values of p<0.05 are listed in the table. DAmP strains are marked in bold.

Interestingly, the GO analysis showed temperature-dependent differences: the 30°C screen revealed chromatin remodeling and histone exchange to be involved in survival upon APAP induced stress, whereas the 37°C screen showed protein localization and vesicle transport to be essential for survival upon APAP induced stress (Table 1).

Furthermore, we investigated whether the enrichment of the resistant strains was effected by the choice of elevated temperature. For this purpose we analyzed a plate grown at 30°C at 100 mM APAP and compared it to a plate grown at 37°C at 70 mM APAP that showed the closest similarity in growth properties (S1 Text, S6 Table). For each plate GO analysis was performed on 179 strains that showed increased growth when compared to the WT (ratio >1.2). The two plates showed total overlap of 64% in APAP resistant strains. GO analysis revealed that genes annotated to protein ubiquitination, RNA polymerase II transcriptional preinitiation complex assembly, mitotic cell cycle phase transition and mitochondria-nucleus signaling pathway were enriched among resistant strains in the both conditions. In addition, the sets of genes driving these enrichments were highly similar between the two conditions (Table A in S1 Text). These results indicate that the identification of ubiquitin-related pathways correlates primarily with exposure to APAP.

### Inactivation of genes involved in protein ubiquitination confers resistance to APAP

The GO enrichment of the resistant strains from the screen identified 18 genes, which were directly or indirectly involved in protein ubiquitination. Also, the deletion strain of *DOA1*, a gene involved in ubiquitin recycling, was identified. Ubiquitination is a posttranslational modification of proteins, which plays an essential role in regulation of various eukaryotic cellular pathways such as protein degradation, DNA repair, vesicular transport, and transcription [34,35]. Seven of the APAP resistant strains were DAmP strains of essential genes: *apc4*-DAmP, *cdc23*-DAmP, *cdc34*-DAmP, *cdc36*-DAmP, *ess1*-DAmP and *rsp5*-DAmP and eleven strains were deletion strains: *Δubc13, Δmms2, Δbmh2, Δcul3, Δupf1, Δupf3, Δubi4, Δhrd1, Δpsh1, Δubc11* and *Δylr224w*. These genes are involved in the regulation of a variety of cellular processes through ubiquitination as described in Table 2. Notably, *UBI4* is the poly-ubiquitin gene, responsible for stress-induced expression of elevated levels of ubiquitin in the cell (37). Resistance of *Δubi4* suggests that ubiquitin deficiency might allow for survival under APAP-induced stress.

**Table 2:** Genes related to protein (de)ubiquitination processes that mediate APAP tolerance as described in the Saccharomyces Genome Database.

Gene	Function
***APC4***	Subunit of the Anaphase-Promoting Complex/Cyclosome (APC/C), a ubiquitin-protein (E3) ligase required for degradation of anaphase inhibitors.
*BMH2*	14-3-3 protein; controls proteome at post-transcriptional level, involved in regulation of exocytosis, vesicle transport, Ras/MAPK signaling, and rapamycin-sensitive signaling.
***CDC23***	Subunit of the Anaphase-Promoting Complex/Cyclosome (APC/C), a ubiquitin-protein (E3) ligase required for degradation of anaphase inhibitors.
***CDC34***	Ubiquitin-conjugating enzyme (E2) and catalytic subunit of SCF ubiquitin-protein (E3) ligase complex that regulates cell cycle progression by targeting key substrates for degradation.
***CDC36***	Component of the CCR4-NOT complex, which has multiple roles in regulating mRNA levels. It contributes to ubiquitin-protein transferase activity.
*CUL3*	Ubiquitin-protein (E3) ligase, required for ubiquitin-dependent degradation of the RNA Polymerase II subunit Rpo21.
*DOA 1*	WD repeat protein required for ubiquitin-mediated protein degradation, ubiquitin binding cofactor that complexes with Cdc48p, required for ribophagy, controls cellular ubiquitin concentration.
***ESS1***	Peptidylprolyl-cis/trans-isomerase; regulates phosphorylation of the RNA polymerase II large subunit (Rpo21p) C-terminal domain.
*HRD1*	Ubiquitin-protein (E3) ligase required for endoplasmic reticulum-associated degradation (ERAD) of misfolded proteins.
*MMS2*	Ubiquitin-conjugating (E2) enzyme variant involved in error-free post-replication repair; forms a heteromeric complex with Ubc13.
***MPE1***	Essential conserved subunit of CPF cleavage and polyadenylation factor;; contains a ubiquitin-like (UBL) domain, possible role in ubiquitination of Pap1p.
*PSH1*	E3 ubiquitin ligase targeting centromere-binding protein Cse4p.
***RSP5***	Ubiquitin-protein (E3) ligase involved in ubiquitin-mediated protein degradation; functions in multivesicular body sorting, heat shock response and ubiquitylation of arrested RNAPII.
*UBC11*	Ubiquitin-conjugating (E2) enzyme.
*UBC13*	Ubiquitin-conjugating (E2) enzyme involved in error-free DNA post-replication repair; interacts with Mms2.
*UBI4*	Ubiquitin, encoded as a polyubiquitin precursor.
*UCC1*	F-box protein and component of SCF ubiquitin E3 ligase complexes.
*UPF1*	ATP-dependent RNA helicase of the SFI superfamily involved in nonsense mediated mRNA decay. Reported E3 ligase via its association with Upf3.
*UPF3*	Component of the nonsense-mediated mRNA decay (NMD) pathway, along with (E3) Upf1.

Note: DAmP strains are presented in bold.

### Ubiquitin deficiency confers resistance to APAP

**Fig 2:**
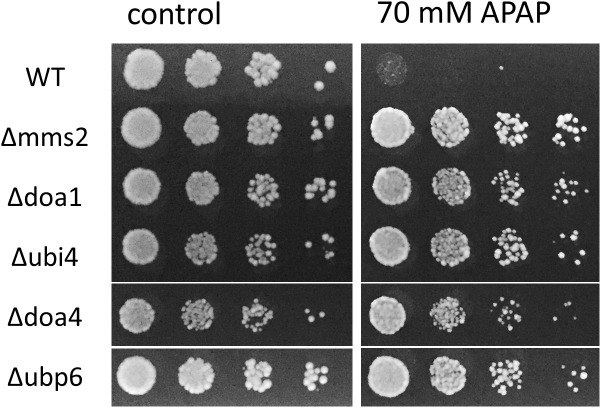
APAP resistance of ubiquitin deficient strains. Five-fold dilution of WT (BY4741), Δ*ubi4*, Δ*doa1*, Δ*ubp6* and Δ*doa4* cells were spotted on YPD plates with or without 70 mM APAP and grown at 37°C for three and four days, respectively.

Two ubiquitin deficient strains, Δ*ubi4* and *Δdoa1*, were identified as APAP resistant. Therefore, we wanted to determine if ubiquitin deficiency was related to APAP resistance. Apart from Δ*ubi4* and *Δdoa1*, two other deletion strains are linked to a reduced level of free ubiquitin in the cell: Δ*ubp6* and Δ*doa4* [36]. Although present in the selective 1522 strains array, they were not detected as significantly different from WT in the screen. Nevertheless, we re-analyzed these two deletion strains for APAP resistance. Our results revealed that these ubiquitin deficient strains were also resistant to APAP when compared to WT, reinforcing the conclusion that ubiquitin deficiency can lead to APAP resistance (Fig 2).

### Ubiquitin overexpression confers sensitivity to APAP

**Fig 3:**
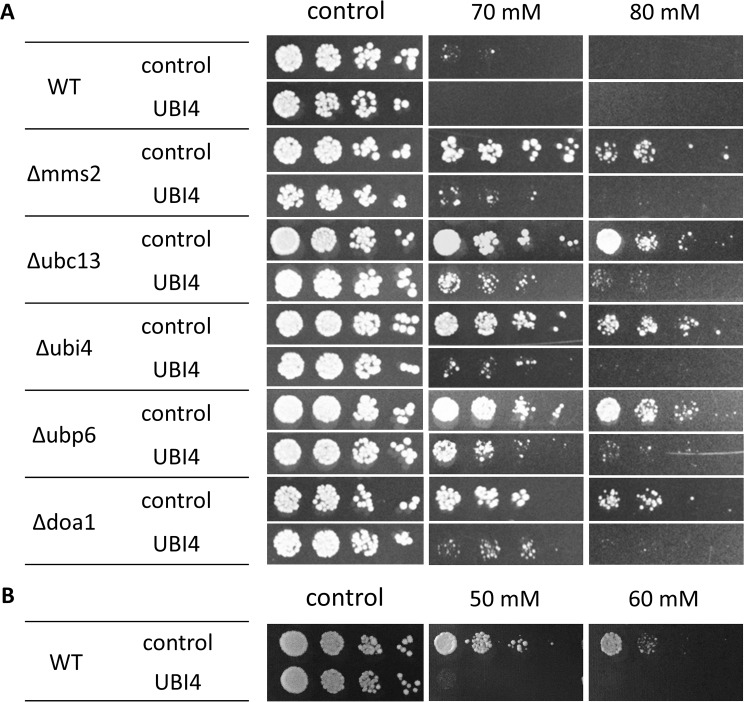
Ubiquitin overexpression confers APAP sensitivity. Strains WT, *Δmms2, Δubc13, Δubi4, Δubp6* and *Δdoa1* were transformed with a multicopy plasmid expressing a ubiquitin gene from the *UBI4* promoter and an empty plasmid as a control. A spotting assay was performed on YPD plates containing 70, 80 and 90 mM APAP (A). A spotting assay performed with WT cells on YPD plates containing 50 and 60 mM APAP (B). YPD plates without APAP were the control. The plates were incubated at 37°C for 3 days for APAP containing plates and 2 days for the control plate. All strains exhibited higher sensitivity to APAP upon ubiquitin overexpression.

The finding that ubiquitin deficient strains are resistant to APAP, suggests that enhanced levels of ubiquitin may cause an increased APAP-sensitivity. To test this, we transformed a multicopy plasmid expressing *UBI4* into WT and the APAP resistant strains Δ*mms2*, Δ*ubc13*, Δ*ubi4*, Δ*ubp6* and Δ*doa1*. Elevated levels of free ubiquitin were confirmed by Western blotting (data not shown). We performed a spot dilution assay on the YPD plates containing three different concentrations APAP (0, 70, 80 mM). Indeed, ubiquitin overexpression resulted in increased sensitivity to APAP, i.e. it suppressed the resistance phenotype of this set of mutants (Fig 3A). Furthermore, a spot dilution assay with lower APAP concentrations 50 and 60 mM, at which WT cells are normally not sensitive, also showed that ubiquitin overexpression increased sensitivity of WT cells to APAP (Fig 3B). Therefore, we conclude that cellular levels of ubiquitin are important for APAP tolerance.

### APAP-induced changes in ubiquitination

**Fig 4:**
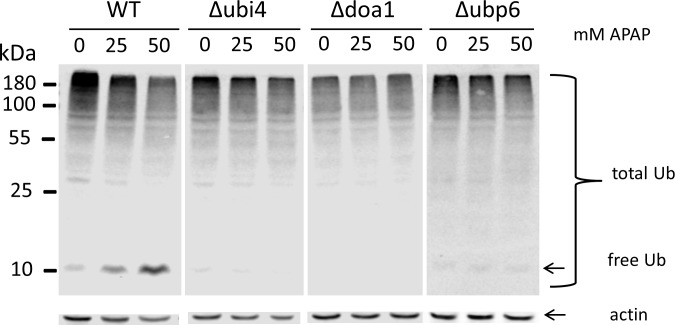
APAP affects levels of free ubiquitin in yeast. The WT, Δ*ubi4*, Δ*doa1* and Δ*ubp6* cells were grown exponentially and treated with 0, 25 or 50 mM APAP for 2 hrs at 37°C before harvesting. 4A) Western blot with antibodies against ubiquitin. 4B) Western blot with anti-actin antibodies was used as an internal control for loading.

Next, we investigated whether APAP exposure has an effect on cellular ubiquitin levels. A Western blot with protein extracts from WT cells, Δ*ubi4*, Δ*doa1*, and Δ*ubp6* strains treated with different concentrations of APAP after prior adaptation to 37°C for 5 hrs was probed with an ubiquitin antibody (Fig 4). A dose-dependent increase in free ubiquitin in WT cells was detected. The Δ*ubi4*, Δ*doa1* and Δ*ubp6* strains showed indeed a reduced level of ubiquitin in the absence of APAP, which was relatively unaffected by APAP exposure. In Δ*doa1*, free ubiquitin levels were below detection level and poly-ubiquitination was relatively unaffected by APAP treatment. Furthermore, the WT strain showed the highest level of (poly)ubiquitination in untreated cells, with a dose-dependent decrease. In contrast, ubiquitin deficient strains Δ*ubi4*, Δ*doa1* and Δ*ubp6* showed lower overall level of (poly)ubiquitination with no or a modest dose-dependent decrease.

### Ubiquitin deficiency and other drugs

**Fig 5:**
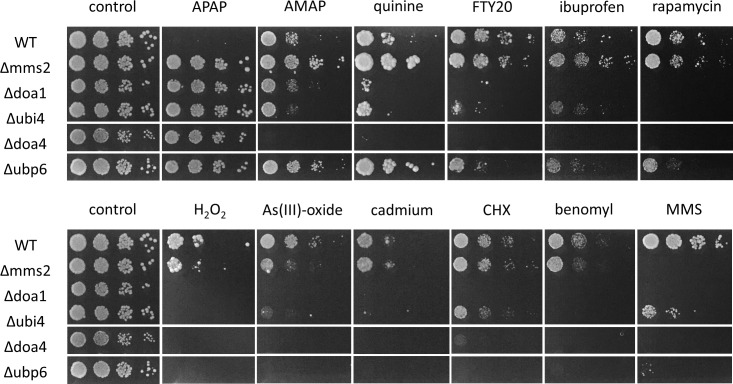
Ubiquitin deficient strains are uniquely APAP resistant and sensitive for a variety of chemicals. The individual dots represent five-fold dilution of the cells: they were spotted on YPD plates with and without the chemicals. The overall conditions were: 70 mM APAP, 90 mM AMAP, 4 mM quinine and 400 μg/μl rapamycin all grown for 5 days; 1 μg/ml cadmium, 0.1 mM arsenic (III)-oxide, 50 mM H_2_O_2_, 0.25 μM cycloheximide (CHX), 30μg/ml benomyl, 0.01% MMS and 15 μM FTY20, all grown for three days; and 2.5 mM ibuprofen grown for 6 days. The plates were incubated at 37°C.

Ubiquitin is generally considered to be required during stress response. Ubiquitin deficiency makes cells susceptible to a variety of chemical and environmental stresses such as heat shock, DNA damage, exposure to heavy metals, protein misfolding, inhibition of translation, and starvation [36–38]. This is in sharp contrast to our observation that ubiquitin deficiency confers resistance to APAP. In order to directly compare the effect of ubiquitin deficiency in yeast on drug sensitivity, WT and the APAP resistant strains Δ*mms*2, Δ*doa1*, Δ*doa4*, Δ*ubp*6 and Δ*ubi4* were treated with APAP, its isomer AMAP and various other chemicals (Fig 5). The spot dilution assay highlights the opposite effect of ubiquitin deficiency, shown by increased resistance to APAP versus enhanced sensitivity towards ibuprofen (analgesic and antipyretic), arsenic trioxide and H_2_O_2_ (oxidative stress), MMS (DNA damage), cadmium (heavy metal) and cycloheximide (translation inhibitor), benomyl (fungicide), quinine (anti-malaria), FTY20 (immunosuppressor) and rapamycin (inhibitor of TOR) in Δ*doa1*, Δ*doa4*, Δ*ubp6* and Δ*ubi4*. Strain *Δmms2* showed enhanced resistance towards APAP, AMAP, and quinine, and sensitivity towards MMS as expected. Notably, AMAP and APAP showed differential toxicity profiles for Δ*ubi4*, Δ*doa1*, Δ*doa4*, but not Δ*ubp6*. In these deletion strains, AMAP was very similar to quinine in its response.

## Discussion

APAP has been marketed for more than 60 years, but a consensus on the mechanism of action is still lacking. Also, potential off-targets involved in APAP toxicity, apart from e.g. NQO2 [17], remain largely unknown. Previously, it was shown that transcription factors Yap1, Yrr1 and Pdr1, all involved in the multidrug response, and the ABC transporter Snq2 play a role in susceptibility to APAP toxicity [15], presumably by lowering the intracellular concentration of APAP by increased efflux. In this study, yeast deletion mutants were chosen to identify genes that play a role in APAP tolerance. The APAP concentrations we used were ~25-50-fold higher than the plasma concentrations reached in patients with an APAP-overdose (1–2 mM) [39]. However, as drug efflux is extremely efficient in yeast, these high concentrations were needed to confer inhibition of growth.

Our study identified several cellular pathways that are involved in APAP toxicity, some of which have been found previously to influence cytotoxicity of other chemicals. Toxicity of APAP is clearly enhanced at higher temperatures. We identified a crucial role for ubiquitin in the toxicity of APAP, both at 30°C and at 37°C. Not only is the cellular concentration of ubiquitin important for APAP tolerance, also various processes controlled by ubiquitination are pivotal. For example, loss-of-function alleles of genes related to ubiquitination, such as the polyubiquitin encoding gene *UBI4*, the E2 Ubiquitin-conjugating enzyme encoding genes *UBC11*, *CDC34*, *MMS2* and *UBC13*, and the E3 Ubiquitin-ligase encoding genes *RSP5* and *HRD1* had an impact on APAP toxicity. Next, we investigated various deletion strains with a known reduced concentration of free ubiquitin, namely Δ*doa1*, Δ*doa4* and Δ*ubp6* [36], which all showed elevated resistance towards APAP. This is in sharp contrast to the common observation that ubiquitin plays a protective role towards drug exposure as apparent in e.g. DNA repair by modification of PCNA [40] and histones [41] or in the removal of unfolded or damaged proteins by the proteasome during stress [37]. Furthermore, GPCRs and transporters are modified by ubiquitin to guide their internalization from the plasma membrane [42].

Ubiquitin is an abundant and highly conserved protein in eukaryotes. Free ubiquitin concentrations are regulated by a variety of regulatory mechanism governed by the expression of precursors fused to ribosomal proteins and of polyubiquitin polypeptides [34,35]. These precursors are cleaved by deubiquitinase enzymes (DUBs) to release ubiquitin monomers. Specialized DUBs exist to control ubiquitin homeostasis by recycling ubiquitin-chains added to proteins channeled to the proteasome or by removing ubiquitin from modified proteins like histones, PCNA and membrane proteins. Our results indicate that the pool of free ubiquitin contributes to the toxicity of APAP. For instance, *Δubi4* confers resistance to APAP, although previous studies showed an enhanced sensitivity of Δ*ubi4* for high temperature, starvation and amino acid analogs [37]. Other examples illustrating the importance of ubiquitin in drug toxicity are the negative effects of loss of the proteasome-associated deubiquitination enzyme Ubp6 on drug sensitivity towards translational inhibitors [36,38]. Deletion of another deubiquitinase gene, *DOA4*, results in increased sensitivity to heat stress, cadmium and canavanine [43]. The ubiquitin-binding protein Doa1 is essential for protection against DNA damaging agents and overexpression of ubiquitin is able to rescue the proper ubiquitination of PCNA, while H2B ubiquitination is strictly dependent on the presence of Doa1 [44]. Notably, while the Ubiquitin Proteasome System is using K48-linked ubiquitin chains, the DNA damage response in metazoans is dependent on K63-linked chains.

The genome-wide loss-of-function screen identified several genes with a strong bias to DNA damage. Ubc13 and Mms2 are involved in the poly-ubiquitination of Pol30, the yeast equivalent of PCNA by the E3 Rad5. PCNA ubiquitination regulates the switch from regular DNA synthesis to translesion synthesis (TLS) or error-free DNA damage tolerance (DDT) during DNA repair [45]. Deletion of *UBC13* prevents the entry of the error-free DDT-phase, allowing TLS error-prone polymerases to introduce mutations as a consequence. However, Δ*rad5* was not resistant. This might be explained by the fact that Rad5 has multiple roles in cellular response to DNA damage during chromosome replication, post replication repair and translation DNA synthesis [46–48]. The subsequent more detailed screen that was focused on nuclear processes, and included mutants of essential genes and multiple concentrations of APAP, did not corroborate DNA repair as a pathway involved in APAP toxicity, consistent with the general safety profile of the drug. However, APAP is reported to effect mitosis and disturb spindles in mammalian cells [14]. Perhaps Mms2 and Ubc13 have another role besides modification of PCNA related to APAP tolerance or ubiquitin homeostasis. For example, absence of Mms2 and Ubc13 might prevent the activation of a cell-cycle arrest induced by APAP.

The cellular processes that were found to be involved in APAP sensitivity or resistance are protein trafficking, chromatin remodeling, and RNA polymerase II transcription, which are all, at least in part, regulated by ubiquitination. Interestingly, deletion of *RTG1*, *RTG2*, *RTG3* and *MKS1* results in resistance to APAP toxicity, suggesting that the mitochondrial retrograde signaling pathway, which is involved in both mitochondrial quality control and nutrient signaling, also plays a role in APAP-induced toxicity [49,50]. Diclofenac, another common analgesic, causes mitochondrial dysfunction by reactive oxygen species (ROS) production through interference with the electron transport chain (ETC) [51]. In contrast, APAP exposure does not cause an increase in ROS in yeast [15]. The APAP-induced growth arrest in WT cells is likely a result of impairment of one or more crucial cellular processes, regulated by ubiquitination, which prevents cell growth but does not kill the cells.

Genes involved in the regulation of transcription are important for the versatility of yeast to respond to stress (Table 1). Especially, deletion of genes encoding components of the Swr1 chromatin remodeling complex [52] were prominent amongst the sensitive strains (*ARP6*, *SWC3*, *SWC5*, *SWR1*, *VPS7*, *VPS72*). Also, deletion of *HTZ1*, encoding histone variant H2AZ and a substrate for Swr1, resulted in a higher APAP sensitivity than WT. These findings suggest that gene induction is vital for survival under APAP-induced stress. However, these findings are not specific for APAP since deletion of genes involved in chromatin modification were also essential for the survival upon arsenic and quinine exposure [22,24].

GO enrichment analysis on biological processes identified genes involved in protein transport and establishment of protein localization, including CVT pathway, protein targeting to vacuole and Golgi, retrograde and vesicle-mediated transport (See Table 1). This might be related to the expression of ABC transporters that modulate extrusion of APAP (15). Also, these processes are often ubiquitin regulated, such as the internalization of membrane proteins internalization by endocytosis [53].

**Fig 6:**
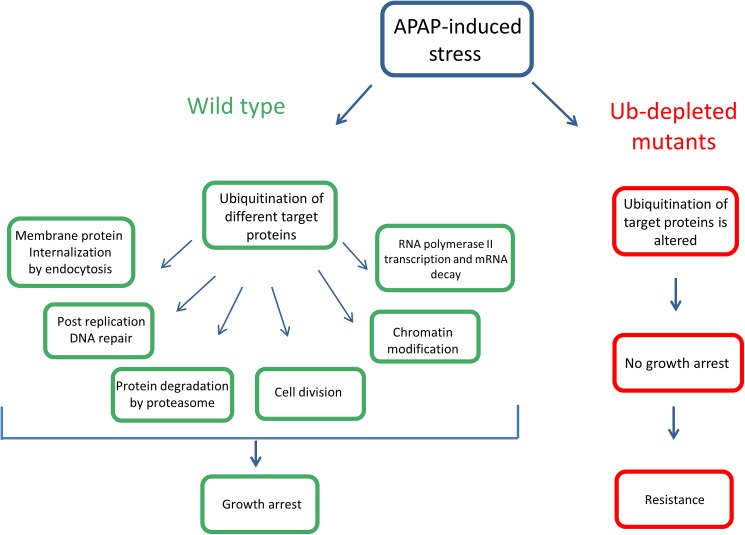
Model to illustrate the effect of APAP on various cellular processes through ubiquitination. In WT cells, the APAP-induced changes in ubiquitination lead to a growth arrest, while in ubiquitin-depleted mutants growth arrest is prevented and cells become resistant.

In conclusion, we found that APAP can influence free ubiquitin levels and our genetic evidence suggests that ubiquitin levels contribute to APAP toxicity, which implies that ubiquitin levels should be considered an important endpoint for drug toxicity studies. Free ubiquitin levels are crucial for the regulation of a wide variety of processes related to protein turnover, endocytosis, DNA repair and chromatin remodeling and transcription (see Fig 6). Recycling of ubiquitin from histones or polyubiquitin chains by deubiquitinating enzymes (like Ubp6 and Doa4) will determine the concentration of free ubiquitin available to properly regulate many cellular processes [54]. APAP-induced stress affects ubiquitination of various target proteins resulting in a reversible growth arrest (Fig 1). Deletion of several E3 Ub ligase genes resulted in resistance, suggesting that the absence of specific protein ubiquitinations correlates with resistance. For instance, several amino acid permeases are rapidly ubiquitinated and degraded upon APAP exposure (manuscript in preparation). Based on our findings, one could design a small set of yeast deletion strains for drug toxicity profiling that (like in Fig 5) might be very useful in classifying drugs and further elucidating the differences in toxicity caused by either APAP or its meta-isomer AMAP [55].

## Supporting information

S1 Fig Complementation assay of APAP resistant strains. Single-copy plasmids containing *MMS2*, *UBC13*, *DOA1*, or *UBP6* were transformed into the corresponding deletion strains, to confirm that the APAP resistance phenotype is due to the deletion of the genes. The individual spots represent a series of five-fold dilutions of the cell cultures, which were spotted on YPD plates with and without 80 mM APAP. The plates were incubated at 37°C for two days.
(TIF)

S2 Fig Complementation assay of APAP sensitive strains. Single-copy plasmids containing HTZ1, GCN5, SWC3 or VPS71 were transformed into the corresponding deletion strains, to confirm that the APAP sensitive phenotype is due to the deletion of the genes. The individual spots represent a series of five-fold dilution of the cells, which were spotted on YPD plates with and without 70 mM APAP. The plates were incubated at 37°C for two days.
(TIF)

S1 Table Genome-wide analysis of APAP resistant mutants. (XLS)

S2 Table List of the 1522 genes included the selective mutant screen. (XLSX)

S3 Table List of APAP resistant strains at 37°C. (XLSX)

S4 Table List of APAP sensitive strains at 30°C. (XLSX)

S5 Table List of APAP sensitive strains at 37°C. (XLSX)

S6 Table List of APAP resistant strains at 30°C. (XLSX)

S1 Text Supporting information. Description of the genome-wide screen for nonessential deletion mutants with altered APAP sensitivity, and the comparison of cell growth at 100mM APAP, 30°C and 70 mM APAP, 37°C.
(DOCX)
