# Supplementary figures and images for: The effect of acetaminophen on ubiquitin homeostasis in Saccharomyces cerevisiae
Source: PLoS One. 2017 Mar 14;12(3):e0173573. doi: 10.1371/journal.pone.0173573 (PMC5349473; doi:10.1371/journal.pone.0173573)

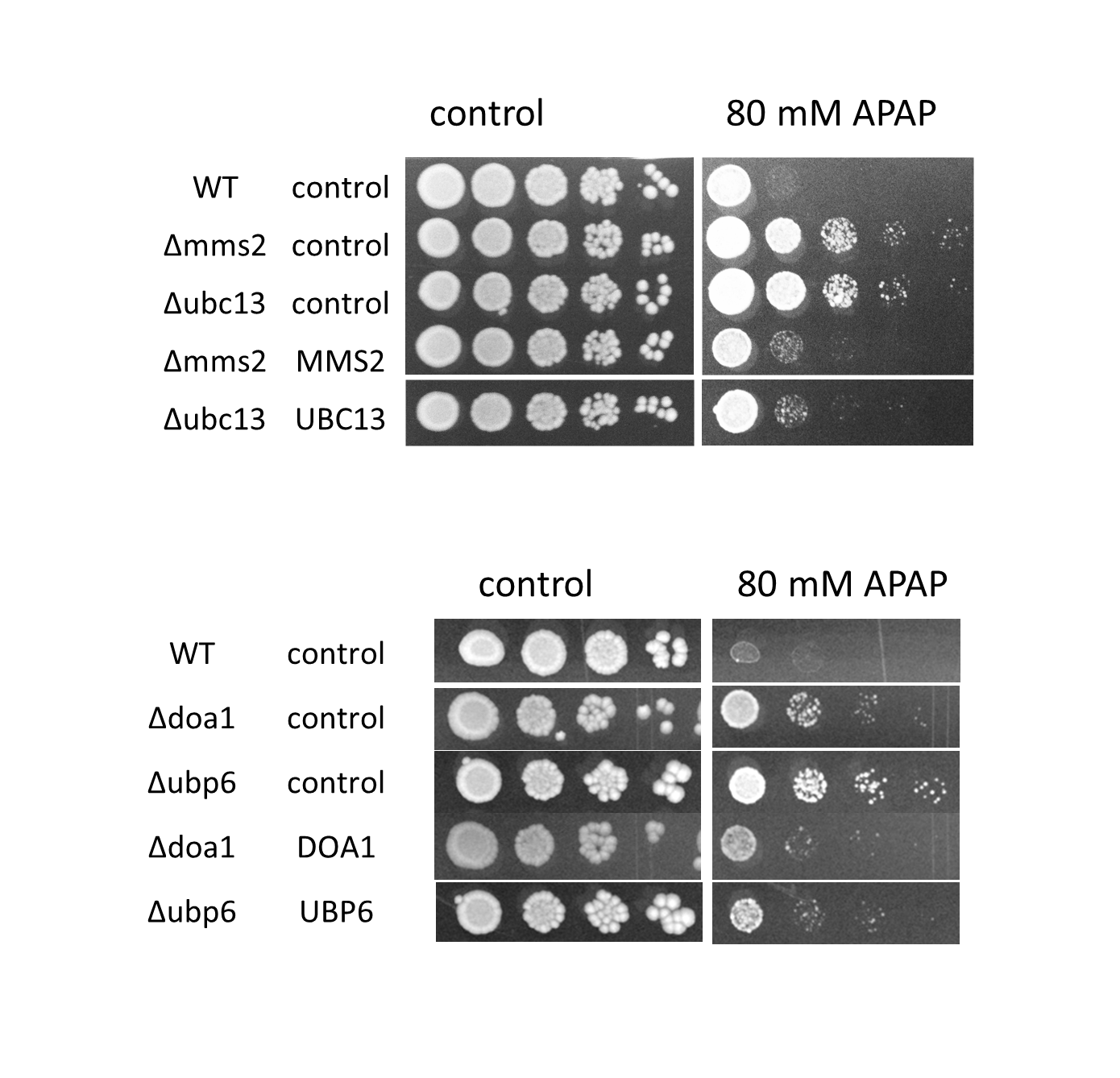

Supplement: S1 Fig — Single-copy plasmids containing MMS2, UBC13, DOA1, or UBP6 were transformed into the corresponding deletion strains, to confirm that the APAP resistance phenotype is due to the deletion of the genes. The individual spots represent a series of five-fold dilutions of the cell cultures, which were spotted on YPD plates with and without 80 mM APAP. The plates were incubated at 37°C for two days. (TIF) [file pone.0173573.s001.tif]

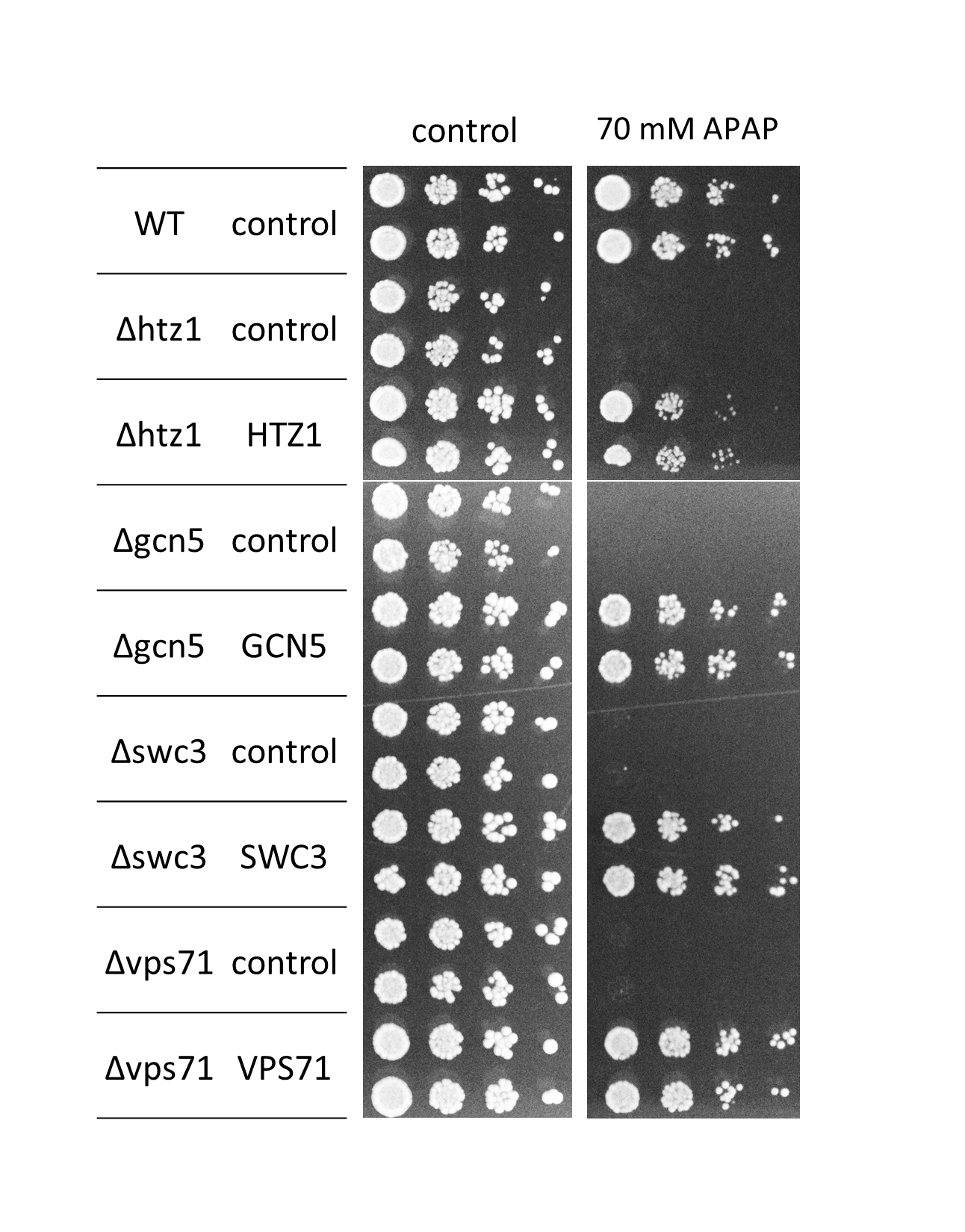

Supplement: S2 Fig — Single-copy plasmids containing HTZ1, GCN5, SWC3 or VPS71 were transformed into the corresponding deletion strains, to confirm that the APAP sensitive phenotype is due to the deletion of the genes. The individual spots represent a series of five-fold dilution of the cells, which were spotted on YPD plates with and without 70 mM APAP. The plates were incubated at 37°C for two days. (TIF) [file pone.0173573.s002.tif]
