# Supplementary material for: The effect of acetaminophen on ubiquitin homeostasis in Saccharomyces cerevisiae
Source: PLoS One. 2017 Mar 14;12(3):e0173573. doi: 10.1371/journal.pone.0173573 (PMC5349473; doi:10.1371/journal.pone.0173573)
Supplement: S1 Text — Description of the genome-wide screen for nonessential deletion mutants with altered APAP sensitivity, and the comparison of cell growth at 100mM APAP, 30°C and 70 mM APAP, 37°C. (DOCX) [file pone.0173573.s009.docx]

# Supporting information

## Genome-wide screen for APAP resistant yeast mutants

### Material and methods

The *Saccharomyces cerevisiae* nonessential gene deletion collection (Open Biosystems, Huntsville, AL) (1) was pinned onto four plates of YEPD containing 16 mg/ml APAP in a format that contains 1536 deletion mutant strains per plate. Plates were grown at 37ºC and photographed with a digital camera (Canon Powershot G5, 5.0 megapixels). Images were converted to greyscale in Photoshop (Adobe) and saved as .tif file. In addition, images were cropped and rotated by Quantity One software (Bio-Rad). Images were automatically loaded, background was corrected and the images were overlaid with a grid to detect colonies in CellProfiler software (2–4). For each colony, the integrated intensity (colony size + intensity) and the area (colony size) were exported to Excel, after which non-growing colonies (area = 1) were removed from the dataset. The average integrated intensity ± standard deviation (SD) was calculated and a confidence interval of 95% was set as average + 2 * SD per plate. Two independent experiments were done with the full deletion mutant collection and analyzed as described above. Deletion mutant strains with an integrated intensity larger than the confidence interval in both experiments were selected for further analysis (see S1 Table). Spot dilution assays were used to verify the resistance of Δ*rfx1*, Δ*ubc13* and Δ*irx1*, as well as Δ*mms2.* Various deletion strains were scored as false positives, because only a small number of cells showed enhanced growth potential on APAP.

### Comparison of APAP screen incubation at 30^o^C and 37^o^C

The screens were performed as described in the Material and methods section. The images of plates grown for 1 day at 30^o^C with 100 mM APAP or at 37^o^C with 70 mM APAP were analyzed using the online application SGAtools (5) . The colony sizes for each strain were quantified and expressed as area pixel number (S6 Table). Each strain was normalized against the average of the four WT colonies on each plate and the first 179 strains with higher growth that WT (approximately 1.2 times) were chosen for gene ontology (GO) analysis for each condition separately, and for the set of strains that showed resistance in both conditions. GO analysis was perform using a web application provided by the SGD database (Table A).

| Table A: APAP resistant strains at 30^o^C 100 mM and 37^o^C 70 mM, and GO enrichment of biological processes in alphabetical order. | | |  |
| --- | --- | --- | --- |
| **Resistant strains at 30^o^C 100 mM APAP**  179/1522 | *AAD4, AIM32, AIR2, ALB1, ANY1, APC1, APC2, APC4, ARO80, BDF2, BLM10, BOI2, BST1, BUB2, BUD3, CAC2, CAP2, CBC2, CDC23, CDC34, CDC36, CDC39, CLB1, CMP2, CPA1, CRN1, CSG2, CTI6, CUL3, DAL81, DBP3, DFG10, DFG16, DMA2, DOT6, DPB2, DPB11, ECM5, ENT4, ESS1, FAR8, FAR10, FPR1, FRE4, FUN30, GAC1, GFD2, GNP1, GPA2, GRX4, GYP8, HAT1, HEK2, HMT1, HNT1, HOS1, HSM3, HUL5, INP51, ISY1, ITC1, IXR1, KAR5, KGD1, LAG1, LIF1, LIN1, LSM7, MAD1, MBP1, MED6, MIG1, MMS2, MNL1, MPE1, MRX1, MSH2, MTC7, NAM7, NCS2, NHP6A, NMD2, NUC1, NUT2, OAF1, OCA1, OCA5, PAC1, PAM17, PBP1, PEA2, PEX22, PEX34, PHB2, PHO84, PHO86, PKH1, POM152, PPH22, PPZ1, PRS5, PSY4, PTK2, PTP1, PUS1, QCR10, RAD51, RAS1, RAS2, REX4, RGR1, RIM8, RIM13, RIM20, RPA14, RPB2, RPB5, RPL8B, RPL16A, RPL34B, RSC4, RSP5, RTG1, RTG2, RVS167, SAP185, SCJ1, SCS3, SCW11, SET4, SHE4, SIP1, SIW14, SKI3, SKI8, SKN7, SLX1, SLX4, SMA2, SNT2, SPC2, SPT2, STB1, STE24, STP1, STP2, STP4, SXM1, TAF7, TAF8, TAF12, TFB3, TFB4, TIR3, TMA23, TPM2, TRM10, TSA1, TSR3, TYR1, UBA3, UBC11, UBC13, UBP5, UBP11, UCC1, UFD2, UGA1, UME1, UPF3, USA1, YCH1, YGL046W, YGL081W, YGR122W, YHP1, YKU70, YOR342C, YPR084W, YSY6* |  |  |
| GO term | Genes | p-value |  |
| Protein ubiquitination  21 out of 101 | *APC1, APC2, APC4, CDC23, CDC34, CDC36, CUL3, DMA2, ESS1, HUL5, MMS2, MPE1, NAM7, PEX22, RSP5, SNT2, UBC11, UBC13, UCC1, UFD2, UPF3* | 6.725E-10 | |
| RNA polymerase II transcriptional pre-initiation complex assembly  7 out of 24 | *DAL81, ESS1, MED6, NHP6A, TAF7, TAF8, TAF12* | 7.612E-04 |  |
| Mitotic cell cycle phase transition  19 out of 97 | *APC1, APC2, APC4, BUB2, CDC23, CDC34, CLB1, DMA2, DPB11, ESS1, GAC1, MAD1, MBP1, PPH22, PTK2, SAP185, STB1, UBC11, YHP1* | 4.686E-07 |  |
| Mitochondria-nucleus signaling pathway  2 out of 5 | *RTG, RTG2* | 1.206E-02 |  |
|  |  |  |  |
| **Resistant strains at 37^o^C 70 mM APAP**  179/1522 | *AAD4, ADD66, AHC1, AIM32, AIR2, ALG3, ALP1, ANY1, APC1, APC2, APC4, APL6, BMH2, BNI4, BNR1, BOI2, BST1, BUD3, BUL2, CAP1, CAP2, CBC2, CDC4, CDC23, CDC34, CDC36, CDC39, CHK1, CLB1, CLB3, CMP2, CPA1, CSG2, CTI6, CUL3, DAL81, DBP3, DFG16, DMA2, DPB2, DPB11, ECM21, ENT4, EOS1, ESS1, FAR3, FAR8, FAT1, FKH1, FUN30, FUS3, FYV8, GAC1, GAL11, GDS1, GEF1, GFD2, GNP1, GRX4, GSH2, GTB1, GUF1, GYP8, HAP5, HAT1, HNT1, HRD1, HSM3, HUL5, IMP2, ISY1, IXR1, JHD2, KAR5, KGD1, LIA1, LIF1, MAD1, MBP1, MED6, MKS1, MMS2, MNL1, MPE1, MRN1, MRX1, MRX11, MSA2, MSH6, MSL1, MTC7, NAM7, NHP6A, NMD2, NUT2, OAF1, OCA1, PAC1, PBP1, PBS2, PEA2, PET18, PEX17, PEX22, PEX34, PHO84, PKH1, PPG1, PRE9, PSY4, PTK2, RAD59, RAS2, REC114, RGR1, RIM13, RIM20, RKR1, RPB2, RPB5, RPL8B, RPL16A, RPS6B, RPS29A, RSP5, RTG1, RTG2, RTG3, RTT10, RVS167, SAK1, SCD6, SCJ1, SCW11, SDS3, SET4, SIW14, SKI2, SKI8, SKN7, SLX1, SLX4, SMA2, SRB4, SSA2, SSK1, STE24, STP1, STP2, STP4, SXM1, TAF7, TAF8, TAF12, TAN1, TDA3, TFB3, TFB4, TIR3, TMA23, TOF1, TOM7, TPM2, TSR3, UBC11, UBC13, UBP1, UBP11, UBR1, UCC1, UPF3, YCH1, YGL081W, YGR122W, YHP1, YMR102C, YNG1, YNR065C, YSY6* |  |  |
| GO term | Genes | p-value |  |
| Protein ubiquitination  25 out of 101 | *APC1, APC2, APC4, BMH2, BUL2, CDC4, CDC23, CDC34, CDC36, CUL3, DMA2, ESS1, HRD1, HUL5, MMS2, MPE1, NAM7, PEX22, RKR1, RSP5, UBC11, UBC13, UBR1, UCC1, UPF3* | 1.60E-04 |  |
| RNA polymerase II transcriptional preinitiation complex assembly  9 out of 24 | *DAL81, ESS1, GAL11, MED6, NHP6A, SRB4, TAF7, TAF8, TAF12* | 1.66E-03 |  |
| Mitotic cell cycle phase transition  20 out of 97 | *APC1, APC2, APC4, CDC4, CDC23, CDC34, CHK1, CLB1, CLB3, DMA2, DPB11, ESS1, FKH1, GAC1, MAD1, MBP1, MSA2, PTK2, UBC11, YHP1* | 5.62E-03 |  |
| Mitochondria-nucleus signaling pathway  4 out of 5 | *MKS1, RTG1 ,RTG2, RTG3* | 7.97E-04 |  |
|  |  |  |  |
| **Resistant strains overlap between 30^o^C and 37^o^C**  114/1522  64% overlap | AAD4, AIM32, AIR2, ANY1, APC1, APC2, APC4, BOI2, BST1, BUD3, CAP2, CBC2, CDC23, CDC34, CDC36, CDC39, CLB1, CMP2, CPA1, CSG2, CTI6, CUL3, DAL81, DBP3, DFG16, DMA2, DPB2, DPB11, ENT4, ESS1, FAR8, FUN30, GAC1, GFD2, GNP1, GRX4, GYP8, HAT1, HNT1, HSM3, HUL5, ISY1, IXR1, KAR5, KGD1, LIF1, MAD1, MBP1, MED6, MMS2, MNL1, MPE1, MRX1, MTC7, NAM7, NHP6A, NMD2, NUT2, OAF1, OCA1, PAC1, PBP1, PEA2, PEX22, PEX34, PHO84, PKH1, PSY4, PTK2, RAS2, RGR1, RIM13, RIM20, RPB2, RPB5, RPL8B, RPL16A, RSP5, RTG1, RTG2, RVS167, SCJ1, SCW11, SET4, SIW14, SKI8, SKN7, SLX1, SLX4, SMA2, STE24, STP1, STP2, STP4, SXM1, TAF7, TAF8, TAF12, TFB3, TFB4, TIR3, TMA23, TPM2, TSR3, UBC11, UBC13, UBP11, UCC1, UPF3, YCH1, YGL081W, YGR122W, YHP1, YSY6 |  |  |
| GO term | Genes | p-value |  |
| Protein ubiquitination  19 out of 104  90% overlap with 30^o^C  75% overlap with 37^o^C | *APC1, APC2, APC4, CDC23, CDC34, CDC36, CLB1, CUL3, DMA2, DPB11, ESS1, GAC1, HUL5, MAD1, MBP1, MMS2, MPE1, NAM7, NMD2, PEX22, PTK2, RSP5, RTG1, RTG2, SKI8, UBC11, UBC13, UCC1, UPF3, YHP1* | 1.02E-04 |  |
| Transcription initiation from RNA polymerase II promoter  7 out of 33  100% overlap with 30^o^C  78% overlap with 37^o^C | *DAL81, ESS1, MED6, NHP6A, TAF7, TAF8, TAF12* | 1.36E-02 |  |
| Mitotic cell cycle phase transition  15 out of 97  79% overlap with 30^o^C  75% overlap with 37^o^C | *APC1, APC2, APC4, CDC23, CDC34, CLB1, DMA2, DPB11, ESS1, GAC1, MAD1, MBP1, PTK2, UBC11, YHP1* | 3.55E-03 |  |
| Mitochondria-nucleus signaling pathway  2 out of 5  100% overlap with 30^o^C  40 % overlap with 37^o^C | *RTG1, RTG2* | 4.64E-02 |  |
| Note: GO enrichment for 30^o^C is calculated against whole genome as background. Only the values of p<0.05 are listed in the table. | | |  |

## References

1. Giaever G, Chu AM, Ni L, Connelly C, Riles L, Véronneau S, et al. Functional profiling of the Saccharomyces cerevisiae genome. Nature [Internet]. 2002 Jul 25 [cited 2016 Aug 1];418(6896):387–91. Available from: http://www.ncbi.nlm.nih.gov/pubmed/12140549

2. Carpenter AE, Jones TR, Lamprecht MR, Clarke C, Kang IH, Friman O, et al. CellProfiler: image analysis software for identifying and quantifying cell phenotypes. Genome Biol [Internet]. 2006 [cited 2016 Aug 1];7(10):R100. Available from: http://www.ncbi.nlm.nih.gov/pubmed/17076895

3. Lamprecht MR, Sabatini DM, Carpenter AE. CellProfiler: free, versatile software for automated biological image analysis. Biotechniques [Internet]. 2007 Jan [cited 2016 Aug 1];42(1):71–5. Available from: http://www.ncbi.nlm.nih.gov/pubmed/17269487

4. Jones TR, Kang IH, Wheeler DB, Lindquist RA, Papallo A, Sabatini DM, et al. CellProfiler Analyst: data exploration and analysis software for complex image-based screens. BMC Bioinformatics [Internet]. 2008 [cited 2016 Aug 1];9:482. Available from: http://www.ncbi.nlm.nih.gov/pubmed/19014601

5. Wagih O, Usaj M, Baryshnikova A, VanderSluis B, Kuzmin E, Costanzo M, et al. SGAtools: one-stop analysis and visualization of array-based genetic interaction screens. Nucleic Acids Res [Internet]. 2013 Jul [cited 2014 May 8];41(Web Server issue):W591–6. Available from: http://www.pubmedcentral.nih.gov/articlerender.fcgi?artid=3692131&tool=pmcentrez&rendertype=abstract
